# Cre-Lox Neurogenetics: 20 Years of Versatile Applications in Brain Research and Counting…

**DOI:** 10.3389/fgene.2016.00019

**Published:** 2016-02-19

**Authors:** Joe Z. Tsien

**Affiliations:** Brain and Behavior Discovery Institute, Medical College of Georgia, Augusta UniversityAugusta, GA, USA

**Keywords:** Cre-loxP, optogenetics, brainbow, mouse brain, learning and memory, neural circuits, cognition, Cre recombinase

## Abstract

Defining and manipulating specific neurons in the brain has garnered enormous interest in recent years, because such an approach is now widely recognized as crucial for deepening our understanding of how the brain works. When I started exploring the Cre-loxP recombination for brain research in the early 1990s, it was written off as a dead-end project by a young fool. Yet over the past 20 years, Cre-lox recombination-mediated neurogenetics has emerged as one of the most powerful and versatile technology platforms for cell-specific gene knockouts, transgenic overexpression, Brainbow imaging, neural pathway tracing with retrovirus and CLARITY, chemical genetics, and optogenetics. Its popularity and greater utility in neuroscience research is also largely thanks to the NIH’s bold *Blueprint for Neuroscience Research Initiative* to launch several Cre-driver resource projects, as well as individual laboratories and private research organizations. With newly-discovered, genetically-encoded molecules that are capable of responding to sonar and magnetic stimulation, for sonogenetics or magnetogenetics, respectively, or detecting rapid voltage changes in neurons, Cre-lox neurogenetics will continue to aid brain research for years to come.

Santiago Ramón y Cajal was among the first to observe and marvel at the elegance and beauty of various neurons in the brain and wonder how they contribute to perception, emotion, memory, and behavior (Cajal, 1909/1910). Structure-functional analysis of neurons began in earnest with the development of single-cell, juxtacellular-labeling, and patch-clamp techniques (Jankowska et al., 1976; McCrea et al., 1976; Hamill et al., 1981; Edwards et al., 1989; Pinault, 1996; Klausberger and Somogyi, 2008). Various other technologies have further transformed the neuroscience landscape at multiple levels – ranging from genes and proteins (Carraway and Leeman, 1973; Hökfelt et al., 1977; Noda et al., 1984; Seeburg et al., 1990; Buck and Axel, 1991; Moriyoshi et al., 1991; Monyer et al., 1992; Carlsson, 1993; Südhof, 2012), to synaptic plasticity and cognitive enhancement (Tang et al., 1999; Lømo, 2003; Frey and Frey, 2008; Wang et al., 2009).

In the late 1980s and early 1990s, following the pioneering efforts by Mario Capecchi, Oliver Smithies, and Martin Evans on gene targeting and embryonic stem cell (ES) technologies (see review by Capecchi, 2005), a small number of laboratories began to generate mutant mice to define genes’ function in development, cancer, or immunology (Zijlstra et al., 1989; DeChiara et al., 1990; Koller et al., 1990; Thomas and Capecchi, 1990). Alcino Silva, Seth Grant, and Thomas O’Dell were among the first to apply the gene knockout approach to study the specific roles of CaMKII or Fyn kinase in plasticity and memory (Grant et al., 1992; Silva et al., 1992a,b). These studies set a new stage to investigate genes and brain functions. However, the limitations such as the lack of phenotypes due to genetic compensation or the developmental effects were noticed. For example, increased spontaneous epilepsy was observed in the CaMKII knockout mice, whereas the dentate gyrus was deformed in the Fyn knockout mice. Both cases led to heated debate in the field about how the reported memory and plasticity deficits should be interpreted. With the NMDA receptor occupying a center stage in plasticity and memory field, Li et al. (1994) set out to demonstrate its role in learning behavior, but they found that NMDAR1 knockout pups all died neonatally due to failed brain development, including the defective suckling reflex. For developmental neuroscientists, it was very exciting, because stereotypic whisker barrel in the thalamus failed to form (Li et al., 1994). Yet for cognitive and behavioral neuroscientists, it was disappointing. It further solidified the argument that deleting the gene in every cell and organ throughout development was not ideal for defining cognitive mechanisms.

## Young and Foolish

My interest in developing the conditional gene knockout technique stemmed from the specific problem at my hand as I was finalizing my HHMI postdoctoral fellowship with Eric Kandel at Columbia University in the summer of 1993. From 1990 to 1993, I was testing the long-held idea, first postulated by Bernie Agranoff (Agranoff and Klinger, 1964), that long-term memory requires new protein synthesis and gene expression. Researchers were racing to identify novel genes whose expressions were regulated by brain activity. We were the first to pull out a set of genes which included tissue-plasminogen activator (tPA), a MAP kinase phosphatase, and a brain-specific immediate early gene BAD1 (published under my former name, Qian et al., 1993, 1994). BAD1 was later also isolated by Paul Worley and named as Arc (Lyford et al., 1995). While isolating these novel genes was exciting, the next logical step for me was to examine their functions in memory. Anti-sense oligonucleotide-based “knock-down” methods were imprecise and not reliable, whereas gene-knockout was a good choice, but its limitations and caveats were obvious.

**FIGURE 1:**
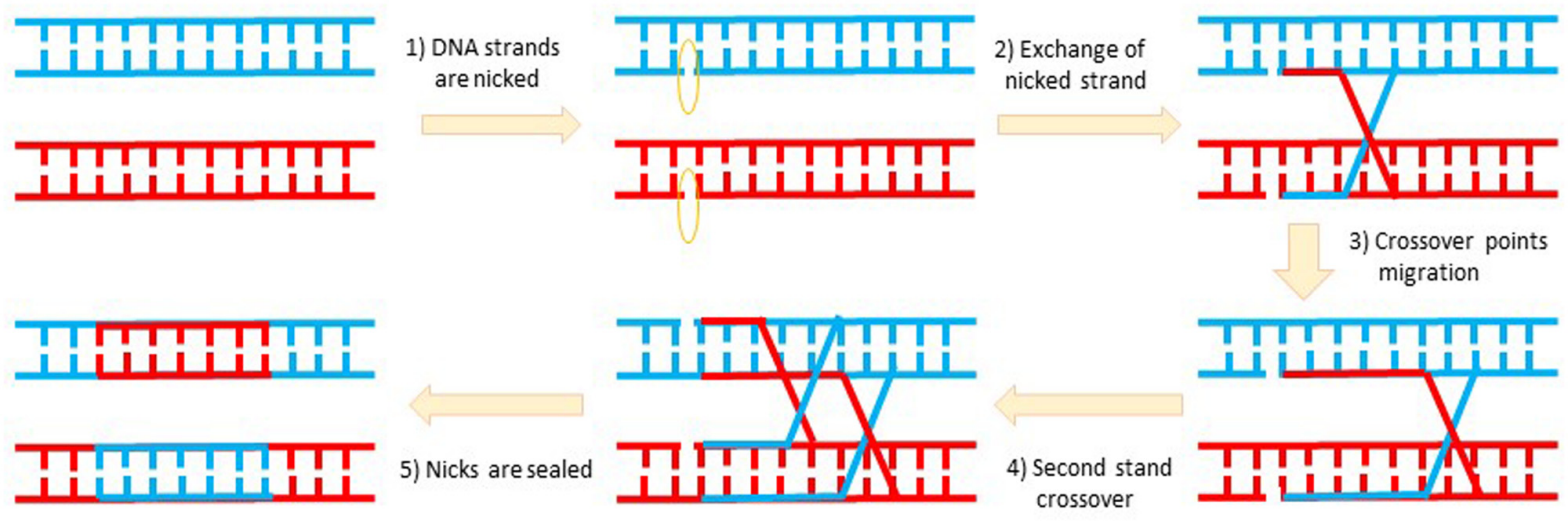
**DNA recombination involves complex topological and biochemical during DNA replication.** DNA recombination is linked with DNA replication during cell divisions in order to exchange genetic materials for the selection of favorable mutations or the elimination of unfavorable or deleterious mutations. Recombination also comes with DNA injury and is therefore tightly coupled with the DNA repair mechanism. Nearly all neurons in the adult brain are post-mitotic to ensure their stability. This explains why brain tumors are in glial cells but not in neurons.

I stumbled upon a paper by Brian Sauer on his successful demonstration of the Cre recombinase-based excision of a floxed marker gene from the circular plasmid transfected in mammalian culture cells (Sauer and Henderson, 1988). The last paragraph of this paper put forth a big question: “*Can Cre also cause recombination at lox sites located within the genome of a mammalian cell?*” This led me to wonder if I could use it to develop brain subregion- and cell type-specific gene knockout and/or transgenic overexpression methods. But I knew that all the textbooks and literature describing DNA replication and recombination go hand in hand with cells division (such as meiosis or mitosis; **Figure 1**). This basic doctrine was imprinted into everyone’s mind, and also evident from the opening sentences of Sauer’s paper: “*The processes governing DNA recombination in mitotic mammalian cells have been the subject of intense investigation in recent years… Mitotic recombination plays a central role in the development and function of the immune system*.” Knowing that all neurons in the adult brain are known to be postmitotic right after birth (except a few in the dentate gyrus and olfactory bulb), any fool who set out to work on DNA recombination in the adult brain would be committing a career suicide. Nature has shown that brain tumors are all in glial cells which divide, but not in neurons. Therefore, DNA recombination as the way to create region- and cell type-specific knockout in the brain was considered to be plainly impossible. I was told that the next show was not about how and why I would fail, but where.

But I was obsessed with the thought of wanting of know the function of genes in a clear way, and only a handful of laboratories had gene-targeting facilities and embryonic stem (ES) cells for making conventional knockout mice. I asked Mario Capecchi of Utah and Susumu Tonegawa of MIT for a second postdoc position, and both said yes. When I sought advice, to my great surprise, Eric suggested that I should choose Susumu’s lab as it would be a better place for trying my idea. I also got the permission to use the CaMKII promoter, cloned by Mark Mayford, which becomes active after the second or third postnatal week and only expresses the forebrain principal cells such as pyramidal cells (Mayford et al., 2005). The stage was all set, with the only minor bug: if my idea did not work out at this second postdoc stage, I would be out of a job.

When I arrived at MIT in the fall of 1993, I was pleased to find that Susumu did not discourage me from exploring Cre-loxP neurogenetics during our 15-s introduction meeting. I was surprised by the zoo-like atmosphere in this 40 postdoctorals/students laboratory. Miraculously, this survival-of-the-fittest model worked well for productivity, and overall MIT was exciting and refreshing to me.

**FIGURE 2:**
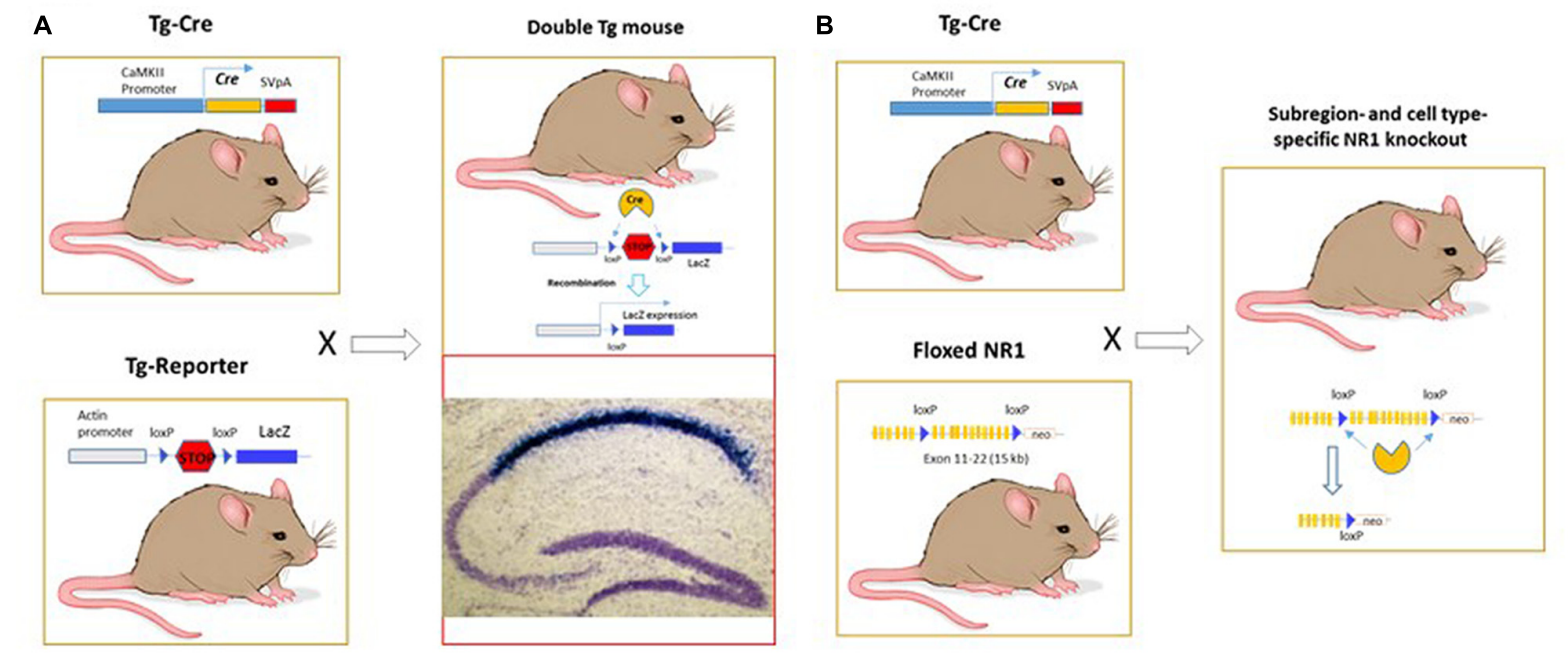
**Cre-LoxP neurogenetics for achieving region- and cell-type specific analysis of relationships of genes, circuits, and functions in the brain. (A)** Strategy to detect the Cre-LoxP recombination in the brain. It required the production of two different transgenic mice: Tg-Cre and Tg-Reporter lines, respectively. These two lines were then crossed to generate the double transgenic mice. CA1-specific recombination was detected after P19 by LacZ method. It is known that CA1 pyramidal cells undergo neurogenesis between E10 and E18 and enter the post-mitotic state by P0. They are well differentiated by P7, with fully established synaptic connections. We have found that Cre-loxP recombination occurs during the middle or end of the third postnatal week in the CA1 pyramidal cells. LacZ in the CA1 pyramidal cells was detected as deep blue color on Nissl stain (in purple-blue) background. **(B)** Conditional knockout of NR1 gene in a specific cell type and region. The exon 11-21 encoding the entire transmembrane domain and C-terminus were flanked by the loxP (termed floxed) in the introns. The second loxP was followed with the neo cassette gene, which allowed for targeted ES cells selection. Luckily, insertion of loxP sites and the neo gene did not alter NR1 gene expression in the floxed homozygous mice.

To develop Cre-loxP-neurogenetics, I must ignore three risks: (1) to hope the textbook on DNA recombination linked with replication was wrong, and my argument was that herpes virus (producing cold sore) infected the peripheral nerve ganglia and somehow replicated itself. (The mechanism still remains unknown to date, but presumably without ganglia neuron division.) (2) The complex procedures and long cycles with no quick feedback and no room for error. I must make various constructs and generate at least three different mouse lines and then embark on a multi-year crossing to breed them together (**Figures 2A,B**). Back then, making even one mutant mouse was already a form of art and luck. In fact, only a few brave souls in well-equipped laboratories could do so because of the complex procedures, lengthy project cycle, expensive cost, and with potentially no phenotypes or undesirable outcomes at the end. A few lucky ones got good jobs and the other unlucky ones simply disappeared. (3) The intentional risk which I brought upon myself: I chose to work on the NMDAR1 as the gene for conditional knockout, instead of the BAD1/Arc. As everyone knew, if the sites to which I inserted the LoxP somehow disrupted its expression, I would have a dead pup that was to be published in a few months by Li et al. (1994). In contrast, unintentional disruption of Arc from the loxP insertion would still give me a conventional knockout paper. But I convinced myself the idea was promising, and my gamble would pay off: I would, at least, get a badge of honor for being *the first* fool to throw myself under the bus of the textbook dogma.

The execution required complex choreography. Despite all of the chaos in the new laboratory, I found friendly colleagues willing to help me. There was David Gerber and Toshikuni Sasaoka who taught me about microinjections in fertilized eggs and blastocysts, respectively; Dongfeng Chen who helped with immune-antibody and staining; Yuqing Li who shared his insights into the NMDAR1 constructs; Min Xu who provided me ES cells and guided me through delicate ES cell-culture procedures; I was also grateful to David Anderson at CalTech for the LacZ reporter and Brian Saucer at Du Pont for Cre-loxP plasmids. I completed all my constructs by January of 1994 and started production of transgenic mice and ES cell targeting of the floxed NR1 construct. I also had a hard-working undergraduate student named Cindy Tom and technician Jason Derwon, who assisted with the genotyping and brain sectioning.

One ominous cloud moved over my head when Klaus Rajewsky reported in July 1994 that they achieved 50% knocked down of the RNA polymerase in T cells (Gu et al., 1994). This meant that even in dividing T cells, Cre-recombination was not efficient: either 50% of the T cells had 100% knockout of RNA polymerase or 100% of the cells had only one copy of the gene deleted, or worse, a mixed mosaic situation. Despite this, I held out the hope (after additional digging into the literature) that the transiently active promoter they used might be the culprit for the poor efficiency during T cell development. Over the ensuing months and years, I stuck my head in the sand and labored over my Cre-loxP neurogenetics experiments. In the late fall of 1994, I finally got the first feedback from the experiments.

On a sunny but cold morning, I recall the great surprise when I saw the intense LacZ staining, specifically in the CA1 pyramidal cells of the hippocampus in the first Cre transgenic line (**Figure 2**). I could not believe my incredible luck, because the CA1 hippocampal region was the center of the universe in the eyes of many plasticity and memory researchers. Additional Cre lines confirmed similar CA1-specific recombination; then other Cre lines showed forebrain-specific patterns. When Susumu returned from a trip to Japan, I told him about what I had found. Once he recovered from his confusion, which seemed to result from jetlag and attempting to grasp all that I had been working on, he overcame his disbelief and immediately shared his jubilation with Alcino over the phone. I asked myself why the CaMKII promoter, which was supposed to express in the forebrain – not specific to the CA1- had such a specific effect. Dongfeng Chen helped provide the clue by revealing that Cre expression was higher in the CA1 pyramidal cells. Over the following 6–8 months, I also obtained the floxed NR1 homozygous mice crossed with the Cre Tg 29.1 line. I confirmed the CA1 pyramidal cell-specific NMDA receptor knockout. Pato Huerta and I proceeded with brain slice recordings or histology, respectively, as soon as we could obtain young adult mice (∼3–5 weeks). We also set aside a set of mice for collaborating with Tom McHugh, Kenny Blum, and Matthew Wilson for place cell recordings in the NR1 mutants. Now, we know that the Cre-lox recombination in T29-1 retained the CA1 specificity only in an age-dependent manner: as Cre expression accumulated over time (starting in the 6th–8th postnatal weeks), it would gradually achieve the forebrain-specific recombination (as expected from the CaMKII promoter).

By the fall of 1996, we were preparing three back-to-back manuscripts to be submitted to *Cell*. We had decided that reporting the feasibility of the Cre-lox neurogenetics would be the first, the CA1-specific NMDA receptor knockout study the second, and the place-cell characterization paper would be published as the third. The original agreement with Eric for using the CaMKII promotor was to include him and Mark as the co-authors in the first paper, in case the Cre-lox work progressed to a point of publication. This initially doomed project now unexpectedly resulted in multiple results, which created its own political nuisance. Knowing the immense interest of the CA1-specific NMDA receptor knockouts in the learning and memory field, Eric preferred his co-authorship be placed in the second paper, not the first Methodology paper. But Susumu insisted that Eric’s name be in the first paper. The back-and-forth conversations that ensued were very distressful to me, but allowed my brain to form an unusually strong long-term semantic memory of what it means to be caught between a rock and a hard place. The papers were eventually published in December 1996 (McHugh et al., 1996; Tsien et al., 1996a,b), but they were left with no corresponding author.

## Cre-Driver Resources for Neurosciences

The successful demonstration of the feasibility of Cre-lox neurogenectis has generated a firestorm in the field. Recognizing its unique usefulness for neuroscience, the NIH launched *the NIH Blueprint for Neuroscience Research*, **known as the Cre-driver project,** to create a collection of mouse strains for better defining the functions of specific cell types and neural circuits in cognitive behaviors^1^. I was glad to provide my inputs during the process. The NIH chose three centers in the United States for the generation of genetically modified mice expressing Cre recombinases in the nervous system. The teams were led by Dr. Ronald Davis at Baylor College of Medicine (now at Scripps Research Institute-Florida), Dr. Josh Huang at Cold Spring Harbor Laboratory (co-led with Sacha Nelson of Brandeis University) and Dr. Ulrich Mueller at Scripps Research Institute. This program generated more than hundreds of Cre-driver lines (Taniguchi et al., 2011). The mice are available through the Mutant Mouse Regional Resource Center (MMRRC) or the Jackson Laboratory’s Cre Repository (see **Table 1**).

**Table 1:** Cre-driver mouse resources.

Cre-driver mice Network	NIH Blueprint for neuroscience	http://www.credrivermice.org
MMRRC (Mutant Mouse Regional Resource Center)		https://www.mmrrc.org/cataloR/StrainCatalogSearchForm.isp
The Jackson Laboratory’s Cre Repository		https://www.jax.org/jax-mice-and-services
The GENSAT project	NIH Blueprint/Rockefeller University	http://www.gensat.org/cre.isp
EMMA (European Mouse Mutant Archive)	European Union, Italy.	www.emmanet.org
MSR (International Mouse Strain Resource)		www.findmice.org
Harwell (Mammalian Genetics Unit)	UK	www.har.mrc.ac.uk
Cre-X-mice Project	Canada	http://nagy.mshri.on.ca/cre_new/index.php
RBRC (RIKEN Biology Resource Center)	Japan	http://mus.brc.riken.jp/en/

The NIH *Neuroscience Blueprint program* also funded the GENSAT project led by Nathaniel Heintz of The Rockefeller University to generate BAC-Cre recombinase driver mouse lines (Gong et al., 2007). A total of 288 Cre lines have been generated to date^2^. Following these efforts, many individual laboratories and institutions also produced a variety of Cre drivers (e.g., Madisen et al., 2010, 2015; Leão et al., 2012). Altogether, at least over 630 Cre lines have been deposited at the Jackson Laboratory to date.

The European Union (EU) also followed the NIH initiative and launched its own Cre-driver project under the name CREATE (Coordination of resources for conditional expression of mutated mouse alleles) (**Table 1**). The CREATE consortium represents a core of major European and international mouse database holders and research groups to develop a strategy for the integration and dissemination of Cre-driver strains for modeling aspects of complex human diseases in the mouse. These Cre lines can be found by contacting EMMA (European Mouse Mutant Archive, Italy) or MSR (International Mouse Strain Resource; **Table 1**). In addition, the United Kingdom, Canada, and Japan have also funded several Cre-driver projects (**Table 1**).

## Cre-Driving Toward Multiple Directions into the Future

In the past decade, one of the most exciting developments is optogenetics which uses light stimulation to manipulate neurons (Zemelman et al., 2002; Boyden et al., 2005). It has been great fun to witness that optogenetics was able to take full advantage of a decade’s investment in generating a rich collection of Cre mouse lines (e.g., CaMKII::Cre, PV::Cre, SOM::Cre, D1: Cre, D2::Cre, ChAT:Cre, or DAT::Cre, etc.), enabling its widespread use in the neuroscience community (Madisen et al., 2012).

However, optogenetics was not the only game in town that benefited from Cre-loxP neurogenetics. Chemical genetics has also emerged as a powerful approach in allowing scientists to activate or suppress specific neurons. For example, one can over-express chemically–genetically modified proteins, such as kinases (Bishop et al., 2000; Wang et al., 2003), or DREADD (Rogan and Roth, 2011) in specific neurons or brain regions. Researchers can also use the floxed diphtheria toxin fragment A (DTA) to delete specific cells using cre lines (Brockschnieder et al., 2004; Matsumura et al., 2004; Buch et al., 2005; Ivanova et al., 2005), and then observe the resulting phenotypes.

In addition, Cre-lox neurogenetics are used to track neural projections in the brain with retrograde virus (Sun et al., 2014; Beier et al., 2015) or CLARITY methods (Lerner et al., 2015). Jeff Lichtman and Joshua Sanes have cleverly explored the unique properties of the Cre-loxP system to randomly express different ratios of red, green, and blue derivatives of green fluorescent protein (GFP) in individual neurons, a technique termed Brainbow (Livet et al., 2007). This allowed them to flag each neuron with a distinctive color. This process has been a major contribution to the field of connectomics.

Cre-lox neurogenetics has also been combined with voltage-sensitive proteins to monitor changes in neuronal activity. Genetically encoded calcium indicators, such as the GCaMPs, have allowed researchers to infer changes in neuron activations via calcium transients (Tian et al., 2009). Researchers are also developing other genetically encoded voltage-sensitive fluorescent proteins that can report cortical electrical responses *in vitro* and/or *in vivo* (Akemann et al., 2010; Ghitani et al., 2015; Madisen et al., 2015).

Most recently, Stuart Ibsen and Sreekanth Chalasani reported an exciting way to use low-pressure ultrasound stimulation to produce microbubbles that amplify the mechanical deformations which can be detected by TRP-4, the pore-forming subunit of a mechanotransduction channel (Ibsen et al., 2015). By overexpressing TRP-4 in specific neurons, the authors showed it can sensitize neurons to an ultrasound stimulus. Similarly exciting, Sheng-Jia Zhang and his colleagues recently reported another noninvasive method, namely magnetogenetics, to activate neurons *in vivo* using magnetic stimulation. The activation of neurons was achieved by neuronal expression of an exogenous magnetoreceptor, an iron–sulfur cluster assembly protein 1 (Isca1) (Long et al., 2015). These two noninvasive approaches offer additional tools to manipulate specific neurons in behaving animals.

Looking back, Cre-lox neurogenetics was written off as a dead-end project, but my intuition and diligence got me through the door. Nowadays, a week rarely goes by without having yet another Cre-based neuroscience finding reported. Cre-lox neurogenetics seems to still have a lot of miles ahead of it. To my students, I always say that being young and foolish can be an asset for the community!

## Author Contributions

The author confirms being the sole contributor of this work and approved it for publication.

## Conflict of Interest Statement

The authors declare that the research was conducted in the absence of any commercial or financial relationships that could be construed as a potential conflict of interest.

## Footnotes

1 http://www.neuroscienceblueprint.nih.gov/factSheet/CreDriver.html

2 http://www.gensat.org/cre.jsp

## References

[B1] Agranoff B. W. Klinger P. D. (1964). Puromycin effect on memory fixation in the goldfish. *Science* 146 952–953. 10.1126/science.146.3646.952 14199725

[B2] Akemann W. Mutoh H. Perron K. Rossier J. Knöpfel T. (2010). Imaging brain electric signals with genetically targeted voltage-sensitive fluorescent proteins. *Nat. Methods* 7 643–649. 10.1038/nmeth.1479 20622860

[B3] Beier K. T. Steinberg E. E. DeLoach K. E. Xie S. Miyamichi K. Schwarz L. (2015). Circuit architecture of VTA dopamine neurons revealed by systematic input-output mapping. *Cell* 162 622–634. 10.1016/j.cell.2015.07.015 26232228 PMC4522312

[B4] Bishop A. C. Ubersax J. A. Petsch D. T. Matheos D. P. Gray N. S. Blethrow J. (2000). A chemical switch for inhibitor-sensitive alleles of any protein kinase. *Nature* 407 395–401. 10.1038/35030148 11014197

[B5] Boyden E. S. Zhang F. Bamberg E. Nagel G. Deisseroth K. (2005). Millisecond-timescale, genetically targeted optical control of neural activity. *Nat. Neurosci.* 8 1263–1268. 10.1038/nn1525 16116447

[B6] Brockschnieder D. Lappe-Siefke C. Goebbels S. Boesl M. R. Nave K. A. Riethmacher D. (2004). Cell depletion due to diphtheria toxin fragment a after Cre-mediated recombination. *Mol. Cell. Biol.* 24 7636–7642. 10.1128/MCB.24.17.7636-7642.2004 15314171 PMC506983

[B7] Buch T. Heppner F. L. Tertilt C. Heinen T. J. Kremer M. Wunderlich F. T. (2005). Cre-inducible diphtheria toxin receptor mediates cell lineage ablation after toxin administration. *Nat. Methods* 2 419–426. 10.1038/nmeth762 15908920

[B8] Buck L. Axel R. (1991). A novel multigene family may encode odorant receptors: a molecular basis for odor recognition. *Cell* 1991 175–187. 10.1016/0092-8674(91)90418-X 1840504

[B9] Cajal S. R. (1909/1910). *Histology of the Nervous System* (trans. Swanson N. Swanson L. W.). New York, NY: Oxford University Press.

[B10] Capecchi M. R. (2005). Gene targeting in mice: functional analysis of the mammalian genome for the twenty-first century. *Nat. Rev. Genet.* 6 507–512. 10.1038/nrg1619 15931173

[B11] Carlsson A. (1993). Thirty years of dopamine research. *Adv. Neurol.* 60 1–10. 8093570

[B12] Carraway R. Leeman S. E. (1973). The isolation of a new hypotensive peptide, neurotensin, from bovine hypothalami. *J. Biol. Chem.* 248 6854–6861. 4745447

[B13] DeChiara T. M. Efstratiadis A. Robertson E. J. (1990). A growth-deficiency phenotype in heterozygous mice carrying an insulin-like growth factor II gene disrupted by targeting. *Nature* 345 78–80. 10.1038/345078a0 2330056

[B14] Edwards F. A. Konnerth A. Sakmann B. Takahashi T. (1989). A thin slice preparation for patch clamp recordings from neurones of the mammalian central nervous system. *Pflugers Arch.* 414 600–612. 10.1007/BF00580998 2780225

[B15] Frey S. Frey J. U. (2008). ‘Synaptic tagging’ and ‘cross-tagging’ and related associative reinforcement processes of functional plasticity as the cellular basis for memory formation. *Prog. Brain Res.* 169 117–143. 10.1016/S0079-6123(07)00007-6 18394471

[B16] Ghitani N. Bayguinov P. O. Ma Y. Jackson M. B. (2015). Single-trial imaging of spikes and synaptic potentials in single neurons in brain slices with genetically encoded hybrid voltage sensor. *J. Neurophysiol.* 2015 1249–1259. 10.1152/jn.00691.2014 25411462 PMC4329433

[B17] Gong S. Doughty M. Harbaugh C. R. Cummins A. Hatten M. E. Heintz N. (2007). Targeting Cre recombinase to specific neuron populations with bacterial artificial chromosome constructs. *J. Neurosci.* 27 9817–9823. 10.1523/JNEUROSCI.2707-07.2007 17855595 PMC6672645

[B18] Grant S. G. O’Dell T. J. Karl K. A. Stein P. L. Soriano P. Kandel E. R. (1992). Impaired long-term potentiation, spatial learning, and hippocampal development in fyn mutant mice. *Science* 258 1903–1910. 10.1126/science.1361685 1361685

[B19] Gu M. Marth J. D. Orban P. C. Mossmann H. Rajewsky K. (1994). Deletion of a DNA polymerase β gene segment in T cells using cell type-specific gene targeting. *Science* 265 103–106. 10.1126/science.8016642 8016642

[B20] Hamill O. P. Marty A. Neher E. Sakmann B. Sigworth F. J. (1981). Improved patch-clamp techniques for high-resolution current recording from cells and cell-free membrane patches. *Pflugers Arch.* 391 85–100. 10.1007/BF00656997 6270629

[B21] Hökfelt T. Elfvin L. G. Elde R. Schultzberg M. Goldstein M. Luft R. (1977). Occurrence of somatostatin-like immunoreactivity in some peripheral sympathetic noradrenergic neurons. *Proc. Natl. Acad. Sci. U.S.A.* 74 3587–3591. 10.1073/pnas.74.8.3587 16592433 PMC431637

[B22] Ibsen S. Tong A. Schutt C. Esener S. Chalasani S. H. (2015). Sonogenetics is a non-invasive approach to activating neurons in *Caenorhabditis elegans*. *Nat. Commun.* 6:8264 10.1038/ncomms9264 PMC4571289 26372413

[B23] Ivanova A. Signore M. Caro N. Greene N. D. E. Copp A. J. Martinez-Barbera J. P. (2005). In vivo genetic ablation by Cre-mediated expression of diphtheria toxin fragment a. *Genesis* 43 129–135. 16267821 10.1002/gene.20162 PMC2233880

[B24] Jankowska E. Rastad J. Westman J. (1976). Intracellular application of horseradish peroxidase and its light and electron microscopical appearance in spinocervical tract cells. *Brain Res.* 105 557–562. 10.1016/0006-8993(76)90603-X 1260464

[B25] Klausberger T. Somogyi P. (2008). Neuronal diversity and temporal dynamics: the unity of hippocampal circuit operations. *Science* 4 53–57. 10.1126/science.1149381 18599766 PMC4487503

[B26] Koller B. H. Marrack P. Kappler J. W. Smithies O. (1990). Normal development of mice deficient in β2M, MHC class I. proteins, and CD8+ T cells. *Science* 248 1227–1230. 10.1126/science.2112266 2112266

[B27] Leão R. N. Mikulovic S. Leão K. E. Munguba H. Gezelius H. Enjin A. (2012). OLM interneurons differentially modulate CA3 and entorhinal inputs to hippocampal CA1 neurons. *Nat. Neurosci.* 15 1524–1530. 10.1038/nn.3235 23042082 PMC3483451

[B28] Lerner T. N. Shilyansky C. Davidson T. J. Evans K. E. Beier K. T. Zalocusky K. A. (2015). Intact-brain analyses reveal distinct information carried by SNc dopamine subcircuits. *Cell* 162 635–647. 10.1016/j.cell.2015.07.014 26232229 PMC4790813

[B29] Li Y. Erzurumlu R. S. Chen C. Jhaveri S. Tonegawa S. (1994). Whisker-related neuronal patterns fail to develop in the trigeminal brainstem nuclei of NMDAR1 knockout mice. *Cell* 76 427–437. 10.1016/0092-8674(94)90108-2 8313466

[B30] Livet J. Weissman T. A. Kang H. Draft R. W. Lu J. Bennis R. A. (2007). Transgenic strategies for combinatorial expression of fluorescent proteins in the nervous system. *Nature* 450 56–62. 10.1038/nature06293 17972876

[B31] Lømo T. (2003). The discovery of long-term potentiation. *Philos. Trans. R. Soc. Lond. B Biol. Sci.* 358 617–620. 10.1098/rstb.2002.1226 12740104 PMC1693150

[B32] Long X. Ye J. Zhao D. Zhang S.-J. (2015). Magnetogenetics: remote non-invasive magnetic activation of neuronal activity with a magnetoreceptor. *Sci. Bull.* 60 2107–2119. 10.1007/s11434-015-0902-0 PMC4692962 26740890

[B33] Lyford G. L. Yamagata K. Kaufmann W. E. Barnes C. A. Sanders L. K. Copeland N. G. (1995). Arc, a growth factor and activity-regulated gene, encodes a novel cytoskeleton-associated protein that is enriched in neuronal dendrites. *Neuron* 14 433–445. 10.1016/0896-6273(95)90299-6 7857651

[B34] Madisen L. Garner A. R. Shimaoka D. Chuong A. S. Klapoetke N. C. Li L. (2015). Transgenic mice for intersectional targeting of neural sensors and effectors with high specificity and performance. *Neuron* 85 942–958. 10.1016/j.neuron.2015.02.022 25741722 PMC4365051

[B35] Madisen L. Mao T. Koch H. Zhuo J. M. Berenyi A. Fujisawa S. (2012). A toolbox of Cre-dependent optogenetic transgenic mice for light-induced activation and silencing. *Nat. Neurosci.* 15 793–802. 10.1038/nn.3078 22446880 PMC3337962

[B36] Madisen L. Zwingman T. A. Sunkin S. M. Oh S. W. Zariwala H. A. Gu H. (2010). A robust and high-throughput Cre reporting and characterization system for the whole mouse brain. *Nat. Neurosci.* 13 133–140. 10.1038/nn.2467 20023653 PMC2840225

[B37] Matsumura H. Hasuwa H. Inoue N. Ikawa M. Okabe M. (2004). Lineage-specific cell disruption in living mice by Cre-mediated expression of diphtheria toxin a chain. *Biochem. Biophys. Res. Commun.* 321 275–279. 10.1016/j.bbrc.2004.06.139 15358172

[B38] Mayford M. Wang J. Kandel E. R. O’Dell T. J. (2005). CaMKII regulates the frequency-response function of hippocampal synapses for the production of both LTD and LTP. *Cell* 81 891–904. 10.1016/0092-8674(95)90009-8 7781066

[B39] McCrea R. A. Bishop G. A. Kitai S. T. (1976). Intracellular staining of purkinje cells and their axons with horseradish peroxidase. *Brain Res.* 118 132–136. 10.1016/0006-8993(76)90847-7 990950

[B40] McHugh T. J. Blum K. I. Tsien J. Z. Tonegawa S. Wilson M. A. (1996). Impaired hippocampal representation of space in CA1-specific NMDAR1 knockout mice. *Cell* 87 1339–1349. 10.1016/S0092-8674(00)81828-0 8980239

[B41] Monyer H. Sprengel R. Schoepfer R. Herb A. Higuchi M. Lomeli H. (1992). Heteromeric NMDA receptors: molecular and functional distinction of subtypes. *Science* 256 1217–1221. 10.1126/science.256.5060.1217 1350383

[B42] Moriyoshi K. Masu M. Ishii T. Shigemoto R. Mizuno N. Nakanishi S. (1991). Molecular cloning and characterization of the rat NMDA receptor. *Nature* 354 31–37. 10.1038/354031a0 1834949

[B43] Noda M. Shimizu S. Tanabe T. Takai T. Kayano T. Ikeda T. (1984). Primary structure of *Electrophorus electricus* sodium channel deduced from cDNA sequence. *Nature* 312 121–127. 10.1038/312121a0 6209577

[B44] Pinault D. (1996). A novel single-cell staining procedure performed in vivo under electrophysiological control: morpho-functional features of juxtacellularly labeled thalamic cells and other central neurons with biocytin or Neurobiotin. *J. Neurosci. Methods* 65 113–136. 10.1016/0165-0270(95)00144-1 8740589

[B45] Qian Z. Gilbert M. Kandel E. R. (1994). Temporal and spatial regulation of the expression of BAD2, a MAP kinase phosphatase, during seizure, kindling, and long-term potentiation. *Learn. Mem.* 1 180–188. 10.1101/lm.1.3.180 10467595

[B46] Qian Z. Tsien J. Z. Gilbert M. E. Colicos M. A. Kandel E. R. Kuhl D. (1993). Tissue-plasminogen activator is induced as an immediate-early gene during seizure, kindling and long-term potentiation. *Nature* 361 453–457. 10.1038/361453a0 8429885

[B47] Rogan S. C. Roth B. L. (2011). Remote control of neuronal signaling. *Pharmacol. Rev.* 63 291–315. 10.1124/pr.110.003020 21415127 PMC3082452

[B48] Sauer B. Henderson N. (1988). Site-specific DNA recombination in mammalian cells by the Cre recombinase of bacteriophage P1. *Proc. Nat. Acad. Sci. U.S.A.* 85 5166–5170. 10.1073/pnas.85.14.5166 PMC281709 2839833

[B49] Seeburg P. H. Wisden W. Verdoorn T. A. Pritchett D. B. Werner P. Herb A. (1990). The GABAA receptor family: molecular and functional diversity. *Cold Spring Harb. Symp. Quant. Biol.* 55 29–40. 10.1101/SQB.1990.055.01.006 1966765

[B50] Silva A. J. Stevens C. F. Tonegawa S. Wang Y. (1992a). Deficient hippocampal long-term potentiation in α-calcium-calmodulin kinase II mutant mice. *Science* 257 201–206. 10.1126/science.1321493 1378648

[B51] Silva A. J. Paylor R. Wehner J. M. Tonegawa S. (1992b). Impaired spatial learning in α-calcium-calmodulin kinase II mutant mice. *Science* 257 206–211. 10.1126/science.1321493 1321493

[B52] Südhof T. C. (2012). Calcium control of neurotransmitter release. *Cold Spring Harb. Perspect. Biol.* 4:a011353 10.1101/cshperspect.a011353 PMC3249630 22068972

[B53] Sun Y. Nguyen A. Q. Nguyen J. P. Le L. Saur D. Choi J. (2014). Cell-type-specific circuit connectivity of hippocampal CA1 revealed through Cre-dependent rabies tracing. *Cell Rep.* 7 269–280. 10.1016/j.celrep.2014.02.030 24656815 PMC3998524

[B54] Tang Y. P. Shimizu E. Dube G. R. Rampon C. Kerchner G. A. Zhuo M. (1999). Genetic enhancement of learning and memory in mice. *Nature* 401 63–69. 10.1038/43432 10485705

[B55] Taniguchi H. He M. Wu P. Kim S. Paik R. Sugino K. (2011). A resource of Cre driver lines for genetic targeting of GABAergic neurons in cerebral cortex. *Neuron* 71 995–1013. 10.1016/j.neuron.2011.07.026 21943598 PMC3779648

[B56] Thomas K. R. Capecchi M. R. (1990). Targeted disruption of the murine int-1 proto-oncogene resulting in severe abnormalities in midbrain and cerebellar development. *Nature* 346 847–850. 10.1038/346847a0 2202907

[B57] Tian L. Hires S. A. Mao T. Huber D. Chiappe M. E. Chalasani S. H. (2009). Imaging neural activity in worms, flies and mice with improved GCaMP calcium indicators. *Nat. Methods* 6 875–881. 10.1038/nmeth.1398 19898485 PMC2858873

[B58] Tsien J. Z. Chen D. F. Gerber D. Tom C. Mercer E. H. Anderson D. J. (1996a). Subregion- and cell type-restricted gene knockout in mouse brain. *Cell* 87 1317–1326. 10.1016/S0092-8674(00)81826-7 8980237

[B59] Tsien J. Z. Huerta P. T. Tonegawa S. (1996b). The essential role of hippocampal CA1 NMDA receptor–dependent synaptic plasticity in spatial memory. *Cell* 87 1327–1338. 10.1016/S0092-8674(00)81827-9 8980238

[B60] Wang D. Cui Z. Zeng Q. Kuang H. Wang L. P. Tsien J. Z. (2009). Genetic enhancement of memory and long-term potentiation but not CA1 long-term depression in NR2B transgenic rats. *PLoS ONE* 4:e7486 10.1371/journal.pone.0007486 PMC2759522 19838302

[B61] Wang H. Shimizu E. Tang Y. P. Cho M. Kyin M. Zuo W. (2003). Inducible protein knockout reveals temporal requirement of CaMKII reactivation for memory consolidation in the brain. *Proc. Natl. Acad. Sci. U.S.A.* 100 4287–4292. 10.1073/pnas.0636870100 12646704 PMC153085

[B62] Zemelman B. V. Lee G. A. Ng M. Miesenböck G. (2002). Selective photostimulation of genetically chARGed neurons. *Neuron* 33 15–22. 10.1016/S0896-6273(01)00574-8 11779476

[B63] Zijlstra M. Li E. Sajjadi F. Subramani S. Jaenisch R. (1989). Germ-line transmission of a disrupted β2-microglobulin gene produced by homologous recombination in embryonic stem cells. *Nature* 342 435–438. 10.1038/342435a0 2685607

